# A Case of Spontaneous Multivessel Coronary Artery Dissection

**DOI:** 10.7759/cureus.34838

**Published:** 2023-02-10

**Authors:** Alexa B Lisevick, Manish Kumar, Asiya Mahmut

**Affiliations:** 1 Surgery, Medical College of Wisconsin, Milwaukee, USA; 2 Cardiology, Pat and Jim Calhoun Cardiology Center, UConn Health, Farmington, USA; 3 Cardiology, Trinity Health of New England, Hartford, USA

**Keywords:** coronary heart disease (chd), younger women, myocardial infarction with no obstructive coronary atherosclerosis, non st-elevation acute coronary syndrome, spontaneous coronary artery dissection

## Abstract

Spontaneous coronary artery dissection (SCAD) is a non-traumatic separation of the epicardial coronary arterial wall leading to luminal obstruction with subsequent myocardial ischemia and infarction. Herein, we describe an interesting case of acute coronary syndrome due to multivessel SCAD without an underlying susceptibility or trigger, and review the literature for SCAD management.

## Introduction

Spontaneous coronary artery dissection (SCAD) is a non-traumatic separation of the epicardial coronary arterial wall occurring via intramural hematoma formation compressing the true lumen, leading to ischemia and acute myocardial infarction [1]. This report presents a compelling case of SCAD with a literature review of the condition.

## Case presentation

A 38-year-old female with a history of hypertension, migraine, obesity (BMI 36 kg/m^2^), and nonischemic cardiomyopathy with recovered ejection fraction (EF) presented to the emergency department (ED) with severe, substernal chest heaviness that started suddenly while she was driving after brushing snow off of her vehicle. She reported accompanying nausea, dyspnea, and diaphoresis. She had two similar prior episodes of mild, self-resolving chest discomfort within the past month.

**Figure 1:**
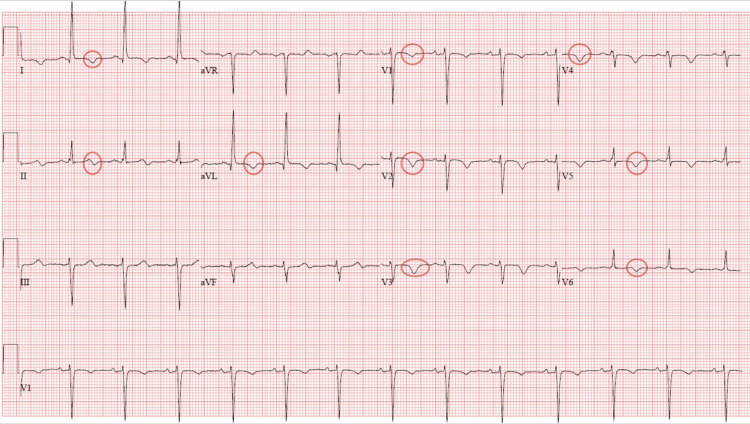
Initial electrocardiogram showing diffuse T wave inversions (red circles) suggestive of myocardial ischemia

Upon arrival at the ED, the patient was asymptomatic and hemodynamically stable. The physical examination was within normal limits. An initial electrocardiogram (ECG) demonstrated normal sinus rhythm with new T wave inversions in V1 through V6 (Figure 1).

**Figure 2:**
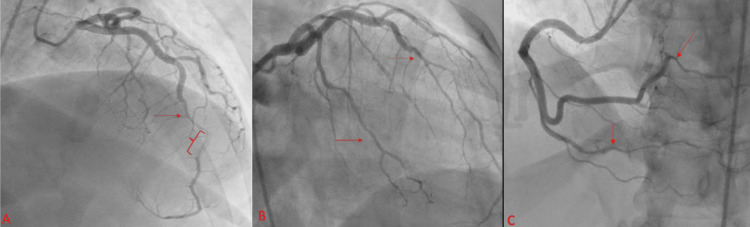
(A) RAO cranial view showing the left anterior descending artery that is initially angiographically normal until the mid-to-distal segment followed by an abrupt change in caliber with a long, smooth tapering progressing to severe diffuse narrowing consistent with SCAD. (B) RAO caudal view showing the smaller first marginal that has severe diffuse narrowing and the second obtuse marginal with moderate diffuse narrowing extending to the distal branches, consistent with diffuse SCAD of the left circumflex system. (C) RAO cranial view showing dissected distal RCA branches including posterior descending artery and posterolateral branches (red arrows denote areas of vessel dissection) RAO, right anterior oblique; SCAD, spontaneous coronary artery dissection; RCA, right coronary artery

Lab work demonstrated a high-sensitivity troponin level of 374 ng/L (normal 0-14 ng/L). While in the ED, she experienced two additional episodes of similar chest discomfort responsive to sublingual nitroglycerin. These episodes were associated with dynamic T wave changes on the ECG. She was given antiplatelet medications, aspirin, and ticagrelor, and heparin infusion for non-ST-segment elevation myocardial infarction (NSTEMI). Because of ongoing pain, a coronary angiogram was done urgently that revealed spontaneous coronary artery dissection of multiple vessels including the mid-left anterior descending (LAD), circumflex obtuse marginal arteries, right marginal artery, and posterior descending artery (Figure 2).

The left ventriculogram showed a left ventricular (LV) EF of 35% with mid to apical anterior, apical, and contiguous apical inferior hypokinesis. Patient's pain had resolved during the procedure and no coronary intervention was done. Heparin was continued for 48 hours in addition to dual antiplatelet therapy (DAPT) and guideline-directed medical therapy (GDMT) for heart failure. Troponin peaked at 1825 ng/L (normal 0-14 ng/L) with a subsequent downtrend. She remained asymptomatic, and was discharged after 72 hours of hospitalization.

## Discussion

SCAD is an acute coronary event in which an intramural hematoma, with or without an intimal tear, leads to the separation of the walls of epicardial coronary arteries [1]. It is a non-traumatic, non-iatrogenic, and non-atherosclerotic cause of acute coronary syndrome (ACS) accounting for 1%-4% of ACS cases overall. It typically affects middle-aged women and younger individuals, as demonstrated in this case; however, SCAD can be seen in patients in their late teens to the ninth decade of life irrespective of underlying coronary atherosclerosis [2,3].

SCAD tends to occur in individuals with pre-existing risk factors, such as fibromuscular dysplasia (FMD), pregnancy and multiparty, connective tissue disorders, exogenous hormone replacement therapy (including oral contraceptives), systemic inflammatory diseases, and migraine headaches [2,3]. A hereditary or genetic component may further account for the predisposition to SCAD [1,4]. Unlike in our case, patients commonly experience a triggering event initiating disruption of the vasa vasorum causing dissection. The triggers are broad, but typically are a source of either extreme physical or emotional stress [1]. Men more commonly report a physical stressor, such as intensive exercise, vomiting, or heavy lifting, whereas women more frequently report an emotionally stressful precipitant [1-3].

Patients with SCAD-driven ACS present similarly to atherosclerotic ACS with characteristic symptoms, ECG changes, and cardiac biomarker elevation. Pregnant patients can have more extensive coronary involvement resulting in severe presentations, such as LV dysfunction, ventricular arrhythmia, or cardiogenic shock due to left main or proximal LAD artery involvement. SCAD has a predilection for the mid to distal coronary arteries, usually involving the LAD artery, though any artery can be affected. Multivessel SCAD can be seen in 9%-23% of the cases, but widespread, diffuse dissection and involvement of distal branches, in the absence of pregnancy, as seen in this case, is rare [2].

Coronary angiography is the gold standard for the diagnosis of SCAD, and three distinct dissection morphologies have been described [2]. The most common, Type 2, appears as a long, smooth narrowing affecting a long segment, usually greater than 20 mm with distal tapering of the termination branches. Type 1 appears as a double radiolucent lumen with arterial wall staining as seen in the LAD artery in this case (Figure 2). Type 3 is focal tubular stenosis similar to atherosclerotic plaque rupture. In cases where the diagnosis is uncertain, noninvasive imaging including optical coherence tomography or intravascular ultrasound can be considered with caution for the risk of propagation of dissection and abrupt vessel closure [1-3].

Acute medical therapy

Extensive evidence-based data for the management of SCAD is lacking; thus, the majority of guidelines are based on expert opinion. The use of heparin and antiplatelet agents in the acute management of SCAD is not well established. However, these agents are routinely used in the management to resolve overlying thrombus at the site of intimal tear balanced against the risk of worsening intramural hematoma and dissection extension. Heparin is usually discontinued once the diagnosis of SCAD is made unless there is an intraluminal thrombus or other indications of systemic anticoagulation. Some experts recommend dual antiplatelet therapy for at least one year following SCAD, especially in those who receive percutaneous coronary intervention (PCI), while others recommend one to three months of DAPT followed by long-term or lifelong aspirin therapy [1,2]. Given the lack of consensus regarding the use and duration of DAPT and aspirin in these patients, further research is needed to assess the risk-benefit of DAPT in this subset of patients. Beta-blockers can be used for LV dysfunction as indicated by the guidelines. Theoretically, beta-blockers reduce shear stress on the coronary arteries and may reduce SCAD recurrence; however, studies are needed for validation and to understand the complex hemodynamic effects on coronary circulation [5].

Revascularization

SCAD lesions heal over time with conservative therapy and recover near-normal coronary architecture; therefore, revascularization is typically not utilized given the risk of dissection propagation and risk of graft or stent failure [3,6]. This approach is in contrast to the invasive revascularization for acute ACS due to plaque rupture or erosion where stenting reduces the risk of recurrence and adverse events [2]. Clinically stable patients with SCAD should be managed conservatively with inpatient monitoring for three to five days to monitor for signs and symptoms of recurrent MI that may occur in 5%-10% of conservatively managed patients, typically within the first seven days [2,7]. Revascularization should be considered in patients experiencing ongoing ischemia, hemodynamic instability, or high-risk anatomies, such as dissection involving the left main or two proximal vessels. PCI in these patients is technically challenging, and often has suboptimal outcomes with a higher rate of complications, such as iatrogenic dissection, abrupt vessel occlusion, hematoma propagation, and strut malposition after healing [1,8]. Urgent coronary artery bypass grafting should be considered in patients with failed PCI, or those with high-risk anatomy [1,2].

Long-term management

The long-term medical management of SCAD should be directed towards the prevention of SCAD recurrence, identifying extra-coronary vascular abnormalities, and improving quality of life. All patients who have ACS should receive cardiac rehabilitation. In consideration of recurrence, patients with hypertension should be closely managed; specifically, beta-blockers may help to reduce SCAD recurrence and should be initiated first [3]. Furthermore, the use of statins is limited given that SCAD is not atherosclerosis mediated; patients who do not have underlying hyperlipidemia are unlikely to benefit from statin therapy. As SCAD may be the first manifestation of underlying vascular abnormalities, patients should receive arterial cross-sectional CT or MR angiography from the brain to the pelvis [1]. Tailored exercise in patients with SCAD is safe and effective, and these patients should be referred for cardiac rehabilitation. SCAD has a recurrence rate of 17%-18% within three to four years and may inflict tremendous psychosocial stress on young patients raising the importance of addressing patient psychosocial well-being. Moreover, female patients should be cautioned about the risks of pregnancy after SCAD and oral contraceptive use; women who wish to become pregnant after SCAD should be referred for preconception counseling [3].

## Conclusions

SCAD is a non-traumatic, non-iatrogenic, and non-atherosclerotic acute coronary event that predominantly affects women and younger patients. While the underlying mechanism is not fully understood, it is traditionally considered to occur in patients with an underlying susceptibility and an inciting event. Diffuse, multivessel SCAD is uncommon, except in pregnant patients where multivessel involvement can be seen. Despite presenting similarly to atherosclerotic-driven ACS, patients with SCAD should be managed conservatively in the vast majority of cases and emphasis should be given to long-term management of this highly recurrent disease.
